# US and UK Consumer Adoption of Cultivated Meat: A Segmentation Study

**DOI:** 10.3390/foods10051050

**Published:** 2021-05-11

**Authors:** Keri Szejda, Christopher J. Bryant, Tessa Urbanovich

**Affiliations:** 1 School of Social and Behavioral Sciences, Arizona State University, Glendale, AZ 85306, USA; 2 Department of Psychology, University of Bath, Bath BA2 7AY, UK; C.J.Bryant@bath.ac.uk; 3 Tessa Urbanovich, MS, Crafton Hills College, Yucaipa, CA 92399, USA; turbanovich@sbccd.cc.ca.us

**Keywords:** cultivated meat, cultured meat, cell-cultured meat, consumer acceptance, consumer adoption, food technology, alternative protein

## Abstract

Despite growing evidence of the environmental and public health threats posed by today’s intensive animal production, consumers in the west remain largely attached to meat. Cultivated meat offers a way to grow meat directly from cells, circumventing these issues as well as the use of animals altogether. The aim of this study was to assess the overall consumer markets and a range of preferences around cultivated meat in the US and the UK relating to nomenclature, genetic modification, health enhancements, and other features. To this end, we recruited large representative samples to participate in an online survey about cultivated meat, and subsequently analyzed segments (a) in the early majority population (guided by the Diffusion of Innovations Model), (b) by generation, and (c) in the general population. Our findings showed a high level of openness (80%) in both the US and UK populations, with 40% somewhat or moderately likely to try and 40% highly likely to try. Younger generations had the greatest openness: 88% of Gen Z, 85% of Millennials, 77% of Gen X, and 72% of Baby Boomers were at least somewhat open to trying cultivated meat. All segments envisioned cultivated meat to be nearly half of their total meat intake. Findings show that consumers prefer the terms ‘cultured’ and ‘cultivated’ over ‘cell-based’ and ‘cell-cultured’ for use in a social context and on packages, even though they perceive these terms as less descriptive. The most important on-package label was one indicating government assurances, and participants preferred non-GM products over GM products. We also found that US consumers prefer nutritionally superior meat over nutritionally equivalent meat. We discuss implications for product development, messaging, and understanding the likely adoption path of this food innovation.

## 1. Introduction

### 1.1. Rationale

Around the world, there are increasingly urgent calls from scientists to drastically reduce global meat consumption [1,2]. While animal products contribute around 57% of food-related greenhouse emissions and use around 83% of the world’s farmland, they provide just 37% of our protein intake and 18% of our calories [3]. A recent study found that moving away from diets heavy in animal products is crucial to staying below 1.5 °C of global temperature increase [4], adding to calls to reduce meat consumption in previous years from the EAT Lancet Commission and the Intergovernmental Panel on Climate Change [1,2]. Moreover, industrial animal agriculture exacerbates public health issues such as antibiotic resistance [5], zoonotic disease [6], and results in the suffering of billions of animals every year [7,8]. Despite these issues, meat consumption rates remain high in developed countries [9].

However, cell culture technology offers the potential to produce meat directly from animal cells rather than rearing live animals. Not only will this technology drastically reduce the need for animals in the production process, it will also decrease greenhouse gas emissions, land use, and water use associated with meat production [10,11,12,13]. Moreover, since cultivated meat can likely be produced in sterile facilities without the use of antibiotics, it can lessen the impact of public health issues commonly associated with conventional meat, including foodborne illnesses [14], pandemic risk [15], and antibiotic resistance [16].

The present study investigated consumer acceptance of cultivated meat in the US and in the UK. These relatively wealthy nations are likely to be key markets for cultivated meat producers, who will initially offer a more expensive product compared to conventional meat. One previous survey has made this comparison, observing that almost 40% of US consumers said that they would eat cultivated meat, compared to just 18% of UK consumers [17]. However, these data are from a non-peer-reviewed industry source and surveyed 615 UK and just 387 US respondents of uncertain representativeness. Therefore, in this study, we will re-examine the acceptance of cultivated meat in the US and the UK to better understand consumers and develop appropriate technologies for a sustainable 2050 protein supply.

### 1.2. The Diffusion of Innovations Model

**Figure 1:**
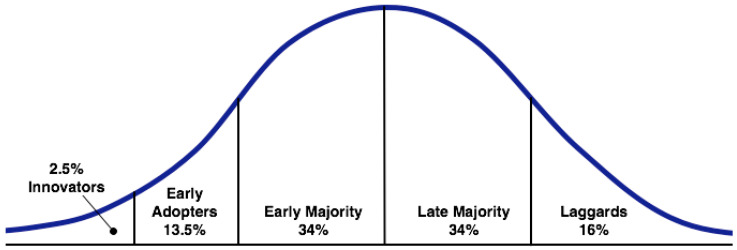
The ‘Diffusion of Innovations’ Model [18,19]. Adopter categorization on the basis of innovativeness. Source: Wikipedia, shared under the Creative Commons Attribution-Share Alike 2.5 License.

Diffusion of Innovations is a framework for examining how new inventions are adopted in the market based on population segmentation [18,19]. Figure 1 shows the model, which separates consumers into five segments based on their openness to new ideas and products. Innovators, who typically make up just 2.5% of the market, are the first people to adopt an innovation. They are eager to use new products and are likely to be willing to pay a premium for the privilege. The next group—called early adopters—make up 13.5% of the market and are the second group to adopt an innovation. They are comfortable with change, need little persuasion to adopt a new product, and are more likely than innovators to provide reliable social proof to the later adopter groups. The early majority and late majority groups each make up 34% of total consumers. These groups are not particularly open to innovations, but are not particularly conservative either. Social proof is important to them—they want to see others using a product and know that it is safe, functional, and beneficial before they adopt it. They may also wait for later iterations of an innovation in which initial problems have been addressed. Finally, the laggards, who make up 16% of the market, are the last to adopt new innovations, and may only do so with significant social coercion.

For stakeholders wishing to accelerate adoption, learning about and appealing to the models’ earlier adoption segments (the innovators, early adopters, and early majority) will be most effective. Consumer segmentation is helpful to identify characteristics of these groups, as well as product and messaging strategies which will be most appealing to them. Meanwhile, companies and organizations need not put much weight on the preferences of laggards, who are unlikely to quickly adopt the new innovation. Therefore, segmenting a market in this way and catering to earlier groups can help to speed up the early stages of an innovation’s acceptance and diffusion through society.

Previous segmentation findings indicate that early adopters hold the same beliefs about cultivated meat as the general population holds on a spectrum of attributes (e.g., health, taste, affordability) [20,21]. However, they tend to hold these beliefs more saliently than the remaining groups do [20].

The present study investigated consumer acceptance of cultivated meat in the United States (US) and the United Kingdom (UK). To obtain a representative sample, our sampling protocol used interlocking sex and age group quotas, as well as region and race/ethnicity quotas. In our assessments, we compared a representative general population sample to an early majority sample, segmenting this group by their reported potential adoption of cultivated meat. In addition, we segmented consumers by generational group (Gen Z, Millennials, Gen X, and Boomers) to identify potential generational differences regarding their support for the technology and their purchase interest. We discuss our segmentation approach in more detail in the Methods section.

### 1.3. Research Questions

#### 1.3.1. Familiarity and Support for the Technology

A number of studies have identified that familiarity with cell-culture technology is a positive predictor of cultivated meat acceptance [22,23,24]. A science communication approach was recently developed for describing the process of producing meat through cellular agriculture in a familiar manner, called ‘meat cultivation’ [25]. We therefore assessed prior familiarity with the technology, as well as support for the technology before and after reading a narrative describing meat cultivation:

RQ1a: What is the current rate of familiarity?
RQ2a: What is the rate of support after reading basic information about the technology?
RQ2b: What is the rate of support after reading educational information about the technology?
RQ2c: How does the level of support change following exposure to a description with a high level of detail about the technology?

#### 1.3.2. Nomenclature

A number of studies have found that nomenclature has a significant impact on cultivated meat acceptance. These studies have largely demonstrated that consumers have a more positive reaction to neutral and benefit-focused terms, in comparison to less appealing terms such as ‘lab grown meat’, which tend to invoke concerns about naturalness [26,27,28]. A recent science communication project assessed nomenclature against several criteria: neutrality, appeal, and descriptiveness, and differentiating from other meat types [25]. In survey studies, ‘cultivated’ meat was found to meet these criteria [25], and in a focus group study, participants assessed these criteria and preferred ‘cultivated meat’ over others [29]. Finally, one study found that ‘cell-based’ met key differentiation criteria for seafood [27]. The most common names currently in use by producers include ‘cultivated meat’, ‘cultured meat’, ‘cell-based meat’.

RQ3a: What are the appeal ratings for each name?
RQ3b: What are the differentiation ratings for each name?
RQ4a: What are the rankings for name preference in a social context?
RQ4b: What are the rankings for name preference on a package label?

#### 1.3.3. Dietary Adoption

There are several studies which investigate the overall acceptance rate of cultivated meat amongst representative national samples. Studies examining US samples have generally indicated that around two thirds of Americans say that they would try cultivated meat, and 25–50% say that they would eat or buy it regularly [23,30,31,32]. Recent studies have also identified solid markets for cultivated meat in Europe, with acceptance rates of around 50–60% in countries including Germany, Italy, and France [24,33]. One previous survey found much higher acceptance of cultivated meat in the US compared to the UK, though this study had some limitations, as discussed above [17]. Studies frequently differentiate between several different measures of acceptance, including willingness to try, buy, use as a replacement for conventional meat, or pay more for cultivated meat [23,32].

RQ5a: How likely are consumers to try cultivated meat?
RQ5b: How likely are consumers to eat cultivated meat as a replacement for conventional meat?
RQ5c: How likely are consumers to purchase cultivated meat regularly?
RQ5d: How likely are consumers to pay a higher price for cultivated meat than conventional meat?
RQ5e: How do consumers estimate their likely percentage of meat intake, in terms of both cultivated and conventional meat?

#### 1.3.4. Benefits

Research on consumer acceptance of cultivated meat has proliferated in recent years [22] and has identified a consistent set of motivators and barriers among consumers. Although consumers generally recognize the benefits of cultivated meat for animals and the environment, many raise taste, price, and safety concerns [21,34]. Some consumers perceive cultivated meat as unnatural, though this depends on how the technology is explained [25,35,36,37]. Disgust and food neophobia are also key barriers for some consumers [23]. A recent study found that personal health benefits of cultivated meat may be most important to consumers. In a survey study of French and German consumers, reduced risk of contamination by pathogens or antibiotics was found to be more persuasive than benefits to animals or the environment [38].

RQ6: What do consumers find as the most important reasons to replace conventional meat with cultivated meat?

#### 1.3.5. Production Preferences

Beyond messaging and nomenclature, there is some evidence that specific product formulations and production methods will impact acceptance. For example, a Dutch study found that small-scale cultivated meat production was more appealing to many [39], while another found that French and German consumers strongly preferred cultivated meat that did not contain genetically modified ingredients over cultivated meat that did [38].

RQ7: Which seals of approval do consumers find important when purchasing cultivated meat?
RQ8: What are consumer preferences in terms of non- or genetically modified cultivated meat products?

#### 1.3.6. Nutrition Preferences

Meanwhile, in a study assessing the possibility of increasing acceptance via nutritional enhancements, the authors found no difference between groups presented with nutritionally enhanced cultivated meat (versus nutritionally equivalent) [40]. However, the researchers noted that other differences in the description may have led consumers to believe that the nutritionally enhanced product would have a less appealing taste. Moreover, recent analysis of the Twitter conversation around cultivated meat revealed that nutritional enhancement was a key positive discussed on cultivated meat [34].

RQ9a: Does the likelihood of purchasing cultivated steak differ depending on whether the product is nutritionally the same, or nutritionally better?

## 2. Materials and Methods

### 2.1. Participants

We obtained ethics approval from the Arizona State University Institutional Review Board (study #00012281) prior to data collection. The study design used a tight sampling protocol to match the adult population aged 18–74 by interlocked sex and age groups to fit within generational groups [41]. Age and sex quotas were established in accordance with population projections [42,43,44]. In addition, we met geographic region and race/ethnicity quotas in the US, and region quotas in the UK. For full demographic data, please view the Supplementary Materials.

The final adult weighted sample size was 4052, with 2018 in the US and 2034 in the UK. We over-recruited (doubled) Gen Z respondents (18–24) to obtain similarly sized groups for each generational category. However, for the main analysis, we applied a 0.5 weight to the 18–24 group (Gen Z). The total sample size reported reflects this weighting; prior to weighting, the actual number of participants was higher (*n* = 2292 for the US and *n* = 2270 for the UK). We used this sampling method to allow for two types of analysis: one based on a representative sample (*n* = 2018, US; *n* = 2034, UK) and another that segmented by generational category (*n* = 2292, US; *n* = 2270, UK). For the generational analysis, we wanted a sufficient sample size for each generational category (i.e., at least 500 in each category). Table 1 below shows the age quotas for the interlocked sex by age groups. Further demographic information including age groups, race/ethnicity, and regions can be found in additional tables in the Supplementary Materials.

The final sample included participants on the basis of the following criteria: survey duration lasting at least five minutes and passing two attention checks. All participants were recruited from CINT panels, and personally identifying information was kept anonymous to the researchers.

### 2.2. Procedures and Materials

#### 2.2.1. Brief Technology Description

After indicating informed consent, participants read a brief, neutral description of the technology that did not use specific nomenclature or describe benefits. Participants were then asked to report their level of support for the technology and their prior familiarity. Participants then rated several names in terms of their appeal and descriptiveness, and then ranked their name preferences in different contexts (social and package label). The specific wording and response options for each 1-item measure are listed below in Table 2.

#### 2.2.2. Expanded Technology Description

In the following section, participants were asked to read an expanded description of the technology. This description used a meat cultivation framework [25] to explain the production process and to describe potential benefits of adopting this technology. The stimuli can be viewed in the Supplemental Materials. Participants then completed outcome measures assessing their level of support, the degree to which they would potentially adopt cultivated meat as a dietary option, reasons for potential adoption, labeling preferences, and preferences regarding use of genetic engineering in the production process. The specific wording and response options for each 1-item measure are listed below in Table 3. 

#### 2.2.3. Nutrition Experiment

In addition to survey questions, we conducted a message design experiment. This was a posttest-only nutrition experiment with random assignment into two experimental groups. In the first condition, the stimulus described the nutrition of cultivated meat as being the same as conventional meat, and in the second condition, the stimulus described the nutrition as better than conventional meat. The dependent variable was the participant’s intent to purchase the cultivated steak product shown in each stimulus.

Participants were then randomly assigned to one of two nutrition experimental groups (RQ9). The stimuli included both a photo of a prepared cultivated steak and a product description that indicated either the same nutritional profile as or a better nutritional profile than conventional meat. Participants then indicated their purchase intention, “How likely are you to purchase this product?” (1 = not at all likely, 5 = extremely likely). Please see Supplementary Materials to view the full stimuli.

#### 2.2.4. Diet and Demographics

In the final part of the survey, participants provided information about their diet and sociodemographics. Dietary information included diet status (vegan, vegetarian, pescatarian, or omnivore) and frequency of meat consumption. Demographic information included age, sex, ethnicity, race, education level, household income, geographic region, and population density at place of residence. Please see the Supplementary Materials to view the demographics tables for the weighted general population sample and the early majority sub-sample. 

### 2.3. Statistical Approach

We specified the hypotheses and analytic plan prior to data collection. Our analysis plan included assessments of both the general population and the early majority group (the group who was very or extremely willing to try cultivated meat) for all questions, as well as generational analysis for support and key dietary adoption measures. Here, we describe the different analyses grouped by type, though the order in which they are reported follows the research questions in Section 1.3.

First, we reported a range of descriptive statistics throughout and a focus on graphic figures to make visual comparisons within a Diffusion of Innovations framework [18,19]. Therefore, we grouped responses by participants’ openness, (i.e., a ‘not at all’ group, a ‘somewhat or moderately’ group, and a ‘very and extremely’ group), and reported the percentage of responses falling within each category. We reported descriptive data in this way for familiarity with cultivated meat, support for cultivated meat before and after reading a pro-cultivated meat narrative, likelihood of trying, buying, and paying more for cultivated meat, as well as using cultivated meat as a replacement for conventional meat. We also report the mean importance scores for different reasons for adopting cultivated meat and different seals of approval for cultivated meat, and the mean expected consumption of conventional and cultivated meat in each country.

Second, we made several comparisons between the US and UK data comparing mean scores using independent samples *t*-tests. We reported these between-country comparisons for the following measures: familiarity with cultivated meat, likelihood of trying, buying, and paying more for cultivated meat, using cultivated meat as a replacement for conventional meat, the importance of different reasons for adopting cultivated meat, the importance of different seals of approval for cultivated meat, the likelihood of buying GM and non-GM cultivated meat.

Third, we used paired samples *t*-tests within countries to test for differences in support for cultivated meat before versus after reading a supportive narrative, and likelihood of buying GM versus non-GM cultivated meat. We report significant effects and effect sizes using Cohen’s d [45], a statistic which reflects the magnitude of small (0.2), medium (0.5), or large (0.8) differences between groups.

Fourth, we used two independent samples *t*-tests to test experimentally purchase intent for nutritionally enhanced versus nutritionally equivalent cultivated meat. Again, we report significant effects and effect sizes using Cohen’s d [45].

Finally, we used one-way ANOVAs to compare the different generational groups on measures of familiarity, support, and dietary adoption. For these analyses, we used unweighted data so that all generational groups contained approximately 500 participants.

## 3. Results and Discussion

### 3.1. Prior Familiarity

This section addresses RQ1 about familiarity. We found that the majority of consumers in both the UK and the US are not at all familiar with cultivated meat currently (54–59%). Slightly more than a third of consumers stated that they were somewhat or moderately familiar (34–41%). Only 7% of US consumers and 5% of UK consumers were very or extremely familiar with the technology.

**Figure 2:**
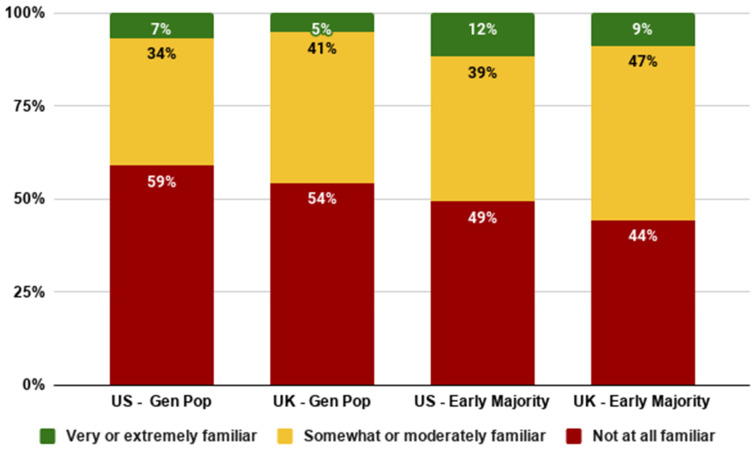
Familiarity with cultivated meat among the general and early majority US and UK populations.

Familiarity was slightly higher among the early majority than the general population (9–12% compared to 5–7%), but still around half of this group had not heard of cultivated meat in both countries. Though this is, as expected, a lower percentage who are completely unfamiliar than the general population, it is interesting to note that half of those who are very or extremely likely to try cultivated meat when they hear about it had no prior familiarity (see Figure 2). This finding underscores the validity of population segmentation based on the Diffusion of Innovation Model: early adopters are open to and supportive of technology about which they are unfamiliar to a greater degree than the overall population is. This finding is also consistent with previous literature showing that prior familiarity is a strong predictor of acceptance [23,24]. Increasing familiarity through various modes of communication will likely be a key catalyst for greater adoption over time.

**Figure 3:**
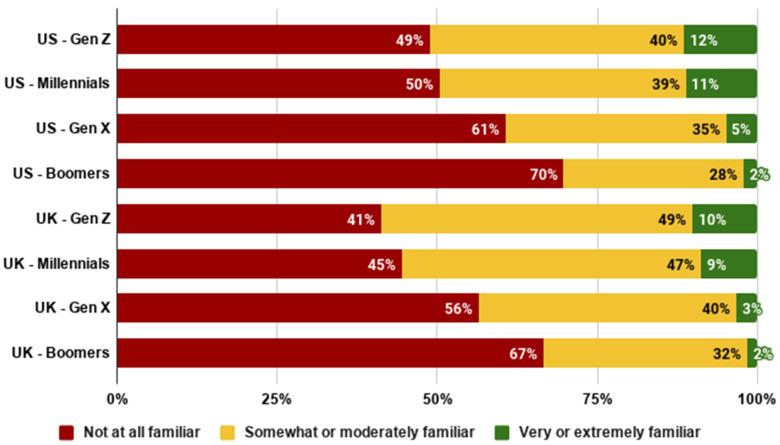
Familiarity with cultivated meat across generations.

As shown in Figure 3, familiarity with cultivated meat was higher amongst younger generations. Over 10% of Gen Z in both countries stated that they were very or extremely familiar with the concept, whilst around two thirds of Boomers were not at all familiar with cultivated meat.

This generational difference was verified using ANOVAs, where the generations were treated as the independent variable and familiarity was the dependent variable. We observed a significant difference in familiarity between the generations in both the US (*F*(3,2288) = 35.923, *p* < 0.001) and the UK (*F*(3,2275) = 49.283, *p* < 0.001).

### 3.2. Support for the Technology

This section addresses RQ2a, RQ2b, and RQ2c about support for cultivated meat. The data showed that before reading the additional supporting narrative, around one quarter of the general population in the UK and the US were very or extremely supportive of cultivated meat: this rose to over 35% after participants read the additional supportive narrative. In both countries, less than 25% were ‘not at all supportive’ of cultivated meat, and this fell to less than 20% after participants read the supporting narrative. Analyses indicated a significant difference between rates of acceptance before and after reading the passage in the US (*t*(2017) = −14.041, *p* < 0.001) and the UK (*t*(2033) = −14.571, *p* < 0.001).

**Figure 4:**
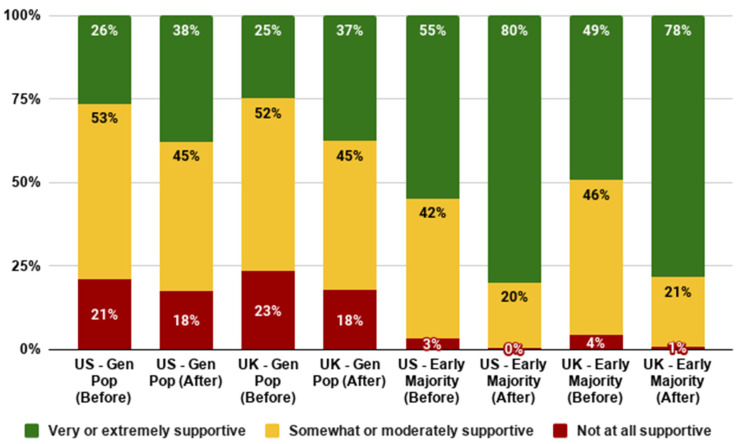
Support for cultivated meat by country and segment.

Overall support was higher amongst the early majority group, as would be expected (Figure 4). Amongst this group, we still see a significant increase in support pre versus post additional information in both the US (*t*(800) = −17.123, *p* < 0.001) and the UK (*t*(802) = −18.202, *p* < 0.001).

**Figure 5:**
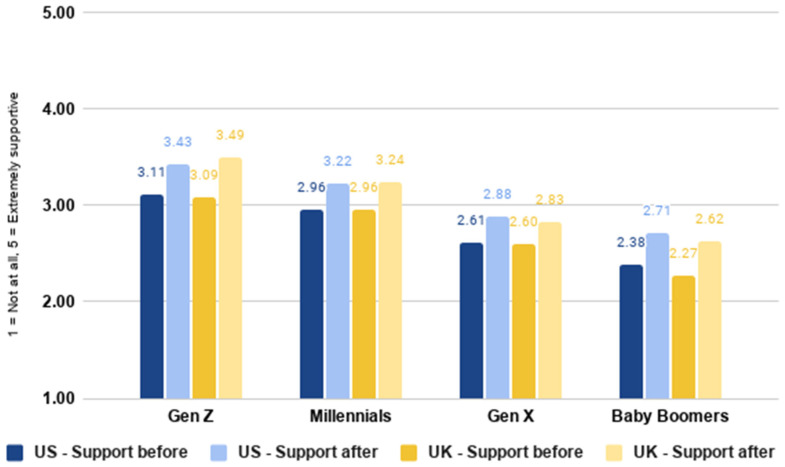
Level of support for cultivated meat before and after expanded technology description by generation.

As shown in Figure 5, younger generations tend to have higher levels of baseline support for cultivated meat. This was the case in both the US (*F*(3,2288) = 42.346, *p* < 0.001) and the UK (*F*(3,2275) = 51.509, *p* < 0.001). Nonetheless, increases in mean levels of support after reading the narrative were observed across all generations and in each country.

### 3.3. Nomenclature Preferences

**Figure 6:**
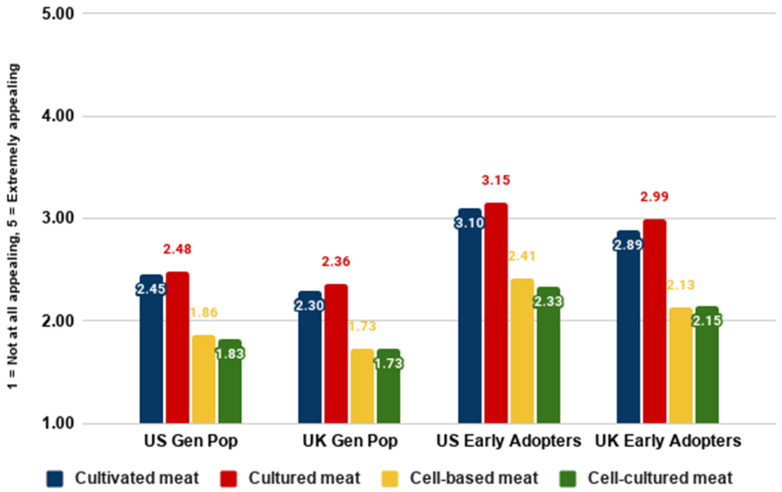
Appeal ratings for different names by country and segment.

**Figure 7:**
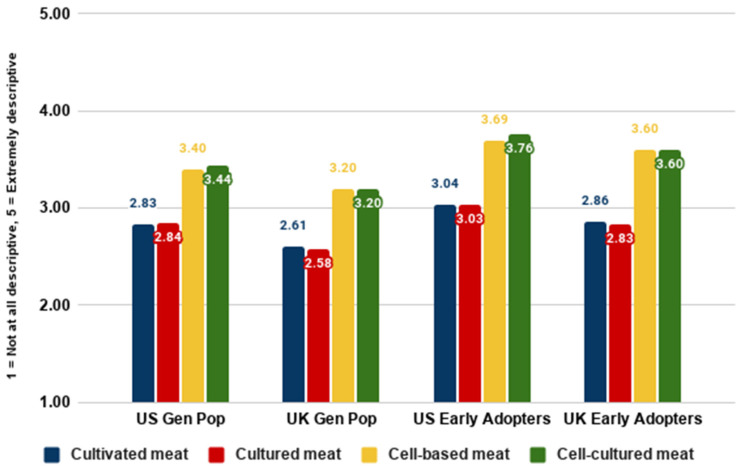
Descriptiveness ratings for different names by country and segment.

Figure 6 and Figure 7 show that the names ‘cultivated meat’ and ‘cultured meat’ were generally perceived as somewhat appealing, while the names ‘cell-based meat’ and ‘cell-cultured meat’ were generally perceived as moderately descriptive. This was generally the case for general population samples as well as the early majority. As well as ratings, we asked participants to rank their preference for different names. In the below graph, lower numbers represent a higher ranking (rank 1 being the most preferred).

This section addresses RQ3a, RQ3b, RQ4a, and RQ4b on nomenclature. Our analyses of different nomenclature indicated that the names ‘cultivated meat’ and ‘cultured meat’ were perceived very similarly to each other, and were generally preferred to the names ‘cell-cultured meat’ and ‘cell-based meat’, which were also perceived very similarly to each other overall. 

**Figure 8:**
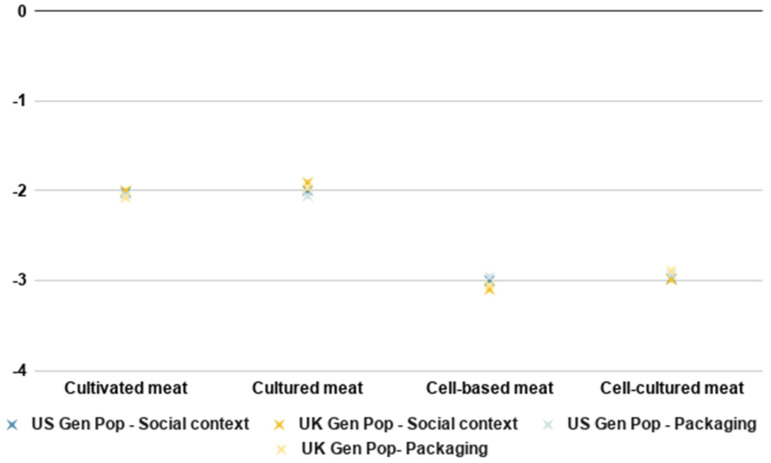
Ranking for different names in a social context and on packaging.

As demonstrated in Figure 8, consumers in both countries tended to prefer the names ‘cultivated meat’ or ‘cultured meat’ over the names ‘cell-based meat’ and ‘cell-cultured meat’ for both a social context and product packaging. Again, the preferences were very similar for both the general population and early majority samples (not shown in Figure 8).

### 3.4. Indicators of Dietary Adoption

Our next set of analyses addressed RQ5a, RQ5b, RQ5c, RQ5d, and RQ5e on dietary adoption. We examined overall indicators of dietary adoption in each country.

**Figure 9:**
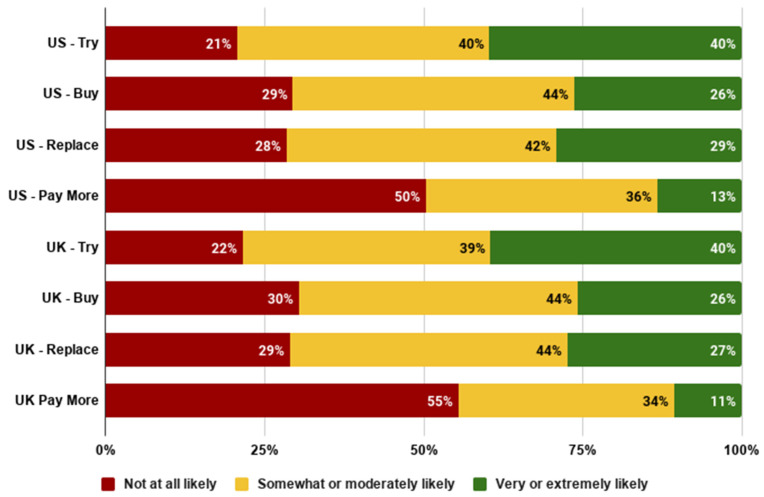
Likelihood of adopting cultivated meat in the US and UK among the general population.

The data indicated that 80% of US and UK consumers were at least somewhat likely to try and 40% of consumers considered themselves very or extremely likely to try cultivated meat (see Figure 9). Around 70% were at least somewhat likely to buy cultivated meat regularly and 25–30% were very or extremely likely to buy regularly or to use it as a replacement for conventional meat. Around half were at least somewhat likely to pay more while 10–13% were very or extremely likely to pay more for cultivated meat.

In addition to looking at the overall rates of acceptance within each country, we used a series of independent samples *t*-tests to compare these measures between the US and the UK (see Table 4).

Our analyses indicated that measures of willingness to buy cultivated meat, and use cultivated meat as a replacement, were not significantly different between the US and the UK samples. However, willingness to pay more for cultivated meat than conventional meat was significantly higher in the US compared to the UK. The same pattern was observed in the general population and the early majority samples. This may be related to the higher average income in the US compared to the UK [46], as well as greater purchasing power [47].

Next, we examined the likelihood of further adoption amongst the early majority sample, which represented the 40% in the US and UK who were very or extremely likely to try cultivated meat.

**Figure 10:**
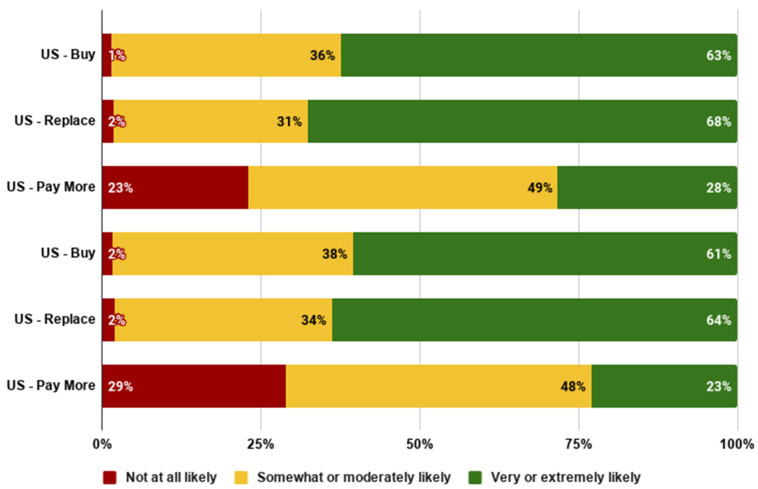
Likelihood of further adoption in the US and UK early majority groups.

Among this early majority samples (see Figure 10), we also found quite high intentions to buy it regularly. Nearly all early majority consumers in both countries were at least somewhat likely to purchase regularly. Over 60% of the early majority were very or extremely likely to buy cultivated meat regularly and to use cultivated meat to replace conventional meat. Nearly three quarters were at least somewhat likely to pay more, and around one quarter were very or extremely likely to pay more.

**Figure 11:**
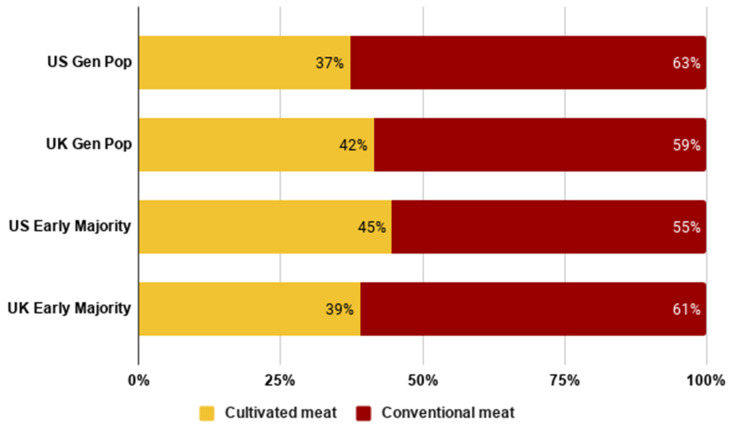
Average expected percentage of meat from each production method by country and segment.

Further, we looked at the early majority’s predicted meat consumption in an imagined future where both cultivated and conventional meat were available, as measured by percentages which indicated the proportion of cultivated and conventional meat that they expected to consume. The results are shown in Figure 11 below.

Results indicate that consumers in both countries expected on average 37–42% of their meat to be coming from cultivated meat. Consumers in the UK expected a slightly higher proportion to be coming from cultivated meat compared to consumers in the US. Generally, consumers envisioned cultivated meat as a partial, rather than full, replacement for conventional meat. 

We also looked at willingness to try and to buy cultivated meat across generations.

**Figure 12:**
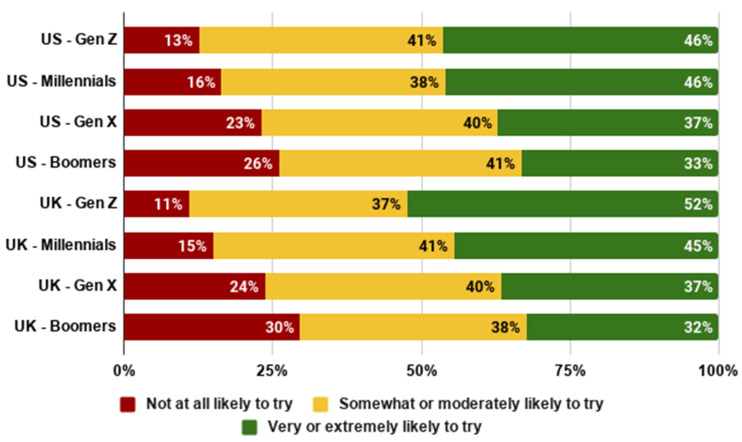
Likelihood of trying cultivated meat by generation.

**Figure 13:**
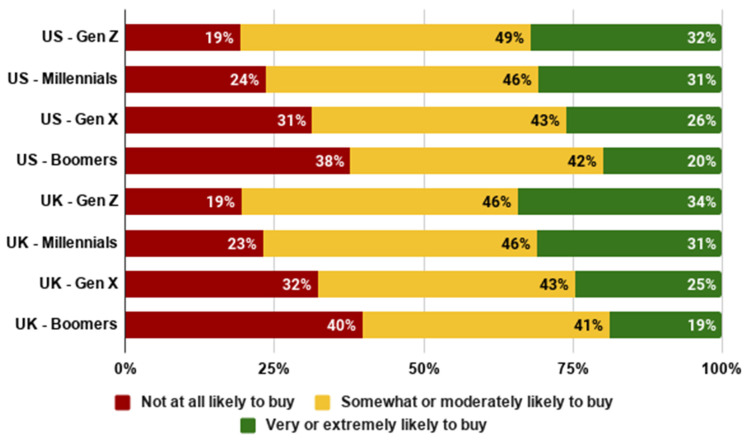
Likelihood of buying cultivated meat by generation.

As shown in Figure 12 and Figure 13, we generally observe that younger generations are more likely to try and to buy cultivated meat. In both the US and the UK, members of Gen Z were the most open to cultivated meat, whilst Baby Boomers were the least open. This is a notable contrast to recent research which was interpreted as suggesting that Generation Z may be particularly closed to cultivated meat [48]. In fact, this study did not compare different generations, and the low acceptance rates observed are more likely a result of methodology, particularly the stimuli presented to participants. Indeed, we observed higher willingness to try amongst younger generations in both the US (*F*(3,2288) = 23.062, *p* < 0.001) and the UK (*F*(3,2275) = 28.590, *p* < 0.001). Similarly, we observed higher willingness to buy amongst younger generations in both the US (*F*(3,2288) = 22.905, *p* < 0.001) and the UK (*F*(3,2275) = 25.057, *p* < 0.001).

### 3.5. Reasons for Dietary Adoption

**Figure 14:**
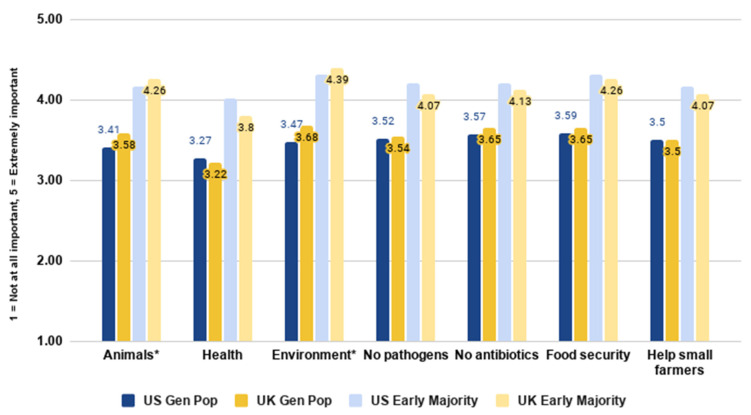
Importance of different reasons for adopting cultivated meat by country and segment.

Our next analysis addressed RQ6, and examined consumers’ reported level of importance of different reasons for adopting cultivated meat. Details are shown in Figure 14 below. 

We observed that cultivated meat being free from pathogens and antibiotics and contributing to global food security were the most important reasons for dietary adoption in the US. In the UK, the most important reasons were cultivated meat being better for the environment, containing no antibiotics, and contributing to global food security. 

Independent samples *t*-tests indicated significant differences between the general populations of the two countries in the importance of the benefits to animals (*t*(4049) = −4.046, *p* < 0.001) and the environment (*t*(4049) = −5.064, *p* < 0.001). In both cases, UK respondents rated these benefits as more important than US respondents.

We also observed that the early majority generally considered all the reasons for adoption to be more important than the general population. Overall, the early majority in both countries considered the environment and global food security important reasons. The US early majority put significantly more importance on health than UK early adopters (*t*(1601) = 4.394, *p* < 0.001). The US early majority also gave significantly higher importance ratings for reasons including containing no pathogens (*t*(1601) = 2.721, *p* = 0.007) and helping small farmers (*t*(1601) = 2.202, *p* = 0.028). Conversely, the UK early majority rated helping animals as significantly more important than those in the US (*t*(1601) = −2.129, *p* = 0.033), reflecting trends in the overall sample. These findings broadly mirrored the findings from a study of French and German consumers, which found that potential health and safety benefits were more important to consumers than ethical or environmental benefits [38].

### 3.6. Preferences for Seals of Approval

Our next set of analyses addressed RQ7, and looked at the relative importance of different assurances which could conceivably appear on cultivated meat labels.

**Figure 15:**
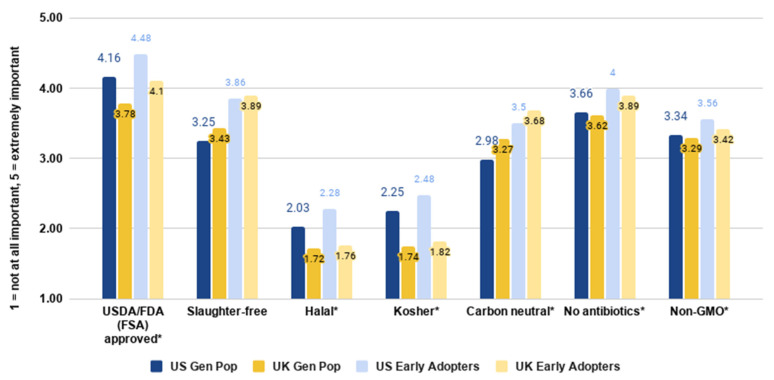
Importance of different seals of approval by country and segment. * indicates a significant difference between the countries on this measure.

As shown in Figure 15, the most important assurance in both countries related to approval by the relevant food safety authority (in the US, this will be the USDA and/or FDA, whereas in the UK, this will be the FSA), followed by the claim that the product is made without antibiotics. This reflects focus group findings which highlighted consumers’ desire for cultivated meat products to be effectively regulated [49].

There were many significant differences between the countries on the importance of these assurances. Independent samples *t*-tests indicated that government-approved (*t*(4049) = 10.119, *p* < 0.001), halal (*t*(4049)=8.016, *p* < 0.001), and kosher (*t*(4049) = 12.652, *p* < 0.001) labels were more important in the US, while slaughter-free (*t*(4049) = −3.991, *p* < 0.001) and carbon-neutral labels (*t*(4049) = −6.893, *p* < 0.001) were more important in the UK. When considering the early majority sample, we also see that antibiotic-free (*t*(1601) = 2.082, *p* = 0.038) and non-GMO (*t*(1601) = 2.153, *p* = 0.031) labels were more important in the US, while the carbon-neutral label only (*t*(1601) = −2.821, *p* = 0.005) was more important in the UK.

Although halal and kosher labels were rated as low importance overall, they were rated as relatively important for Muslim and Jewish respondents, respectively. For Jewish respondents (*n* = 50 US and UK combined), being Kosher was the joint-third most important attribute behind being government-approved and being antibiotic-free, rated equally as important as being non-GMO. For Muslim respondents (*n* = 84 US and UK combined), being Halal was by far the most important assurance, considered more important than being government-approved or antibiotic-free. Scholars have discussed the conditions under which cultivated meat could be called kosher and/or halal, and generally agree that there are viable versions which fit with each of these religions’ requirements [49,50,51]. The present data suggest that Jewish consumers are less concerned with a kosher label than other assurances, while Muslim consumers consider a halal label paramount. Previous research has identified some groups of consumers for whom their food aligning with their culture is extremely important [52], and some survey data have suggested that Muslim consumers are more likely than Jewish consumers to adhere to dietary restrictions (e.g., on pork) [49].

### 3.7. Preferences toward Genetic Engineering

**Figure 16:**
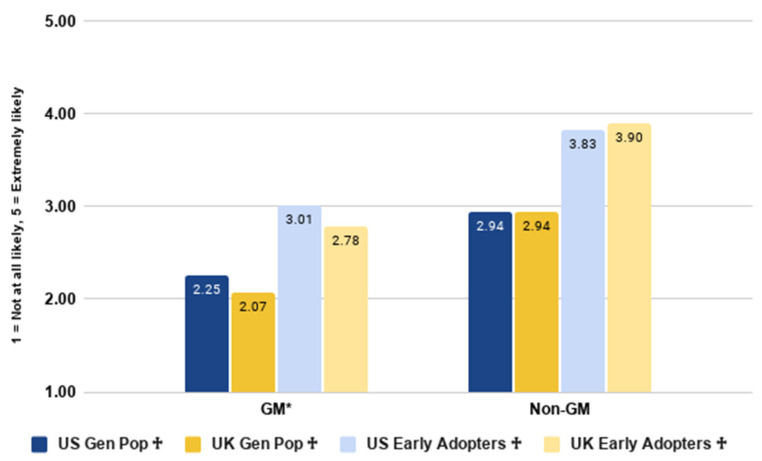
Likelihood of purchase GM and non-GM cultivated meat by country and segment. * indicates a significant difference in purchase intent between the two countries within the identified product. ^†^ indicates a significant difference in purchase intent between the two products within the identified country.

Our next set of analyses addressed RQ8, looking at the difference in willingness to purchase genetically modified or non-genetically modified cultivated meat. All participants indicated their willingness to purchase both types of cultivated meat. Results are shown in Figure 16 below.

Within countries, the difference in purchase intent for GM versus non-GM cultivated meat was tested using two paired samples *t*-tests. Consumers in both the US (*t*(2017) = −21.489, *p* < 0.001) and the UK (*t*(2033) = −28.933, *p* < 0.001) were significantly more willing to purchase non-GM cultivated meat compared to GM cultivated meat. These effect sizes were medium in the US (Cohen’s *d* = 0.537) and in the UK (Cohen’s *d* = 0.685; Cohen, 1992). 

Looking at the early majority sample, both the US (*t*(800) = −13.533, *p* < 0.001) and the UK (*t*(802) = −19.815, *p* < 0.001) were significantly more willing to purchase non-GM cultivated meat compared to GM cultivated meat. This was a medium-to-large effect in the US (Cohen’s *d* = 0.691) and a large effect in the UK (Cohen’s *d* = 0.962) [45]. This mirrors previous findings from Europe showing that consumers prefer non-GM cultivated meat [38].

Further, we used two independent samples *t*-tests to examine differences in purchase intent for either product between countries. There was no significant difference between the two countries in their willingness to purchase non-GM cultivated meat (*t*(4049) = −0.118, *p* = 0.906). However, US respondents were significantly more likely than UK respondents to purchase GM cultivated meat (*t*(4049) = 4.809, *p* < 0.001). This pattern was replicated amongst the early majority sample: there was no significant difference between the two countries in their willingness to purchase non-GM cultivated meat (*t*(1601) = −1.368, *p* = 0.171). However, the US early majority were significantly more likely than the UK early majority to purchase GM cultivated meat (*t*(1601) = 3.621, *p* < 0.001). 

### 3.8. Nutritional Preferences

The next set of analyses examined consumer preferences for a cultivated steak product which was either nutritionally equivalent to conventional meat or nutritionally superior to conventional meat. This was tested experimentally.

**Figure 17:**
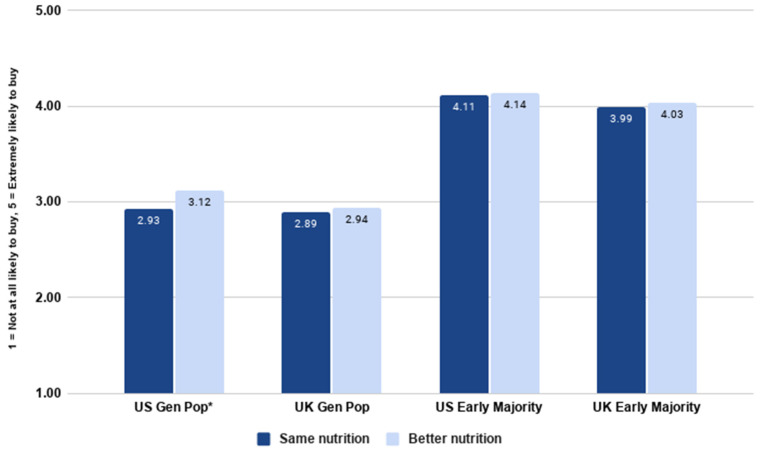
Likelihood of purchasing nutritionally equivalent versus nutritionally enhanced cultivated meat by country and segment. * indicates a significant difference (*p <* 0.05) in purchase intent between the two types of product.

As Figure 17 shows, an independent samples *t*-test indicated that purchase intent was significantly higher for nutritionally superior cultivated meat compared to nutritionally equivalent cultivated meat in the US (*t*(2016) = −3.143, *p* = 0.002). However, in the UK, there was no significant difference in purchase intent between the two products (*t*(2032) = −0.857, *p* = 0.392). Though significant in the US, the effect size was very small (Cohen’s *d* = 0.140), and in the UK, it was almost nonexistent (Cohen’s *d* = 0.037). 

When considering the early majority, there was no significant difference in purchase intent among the early majority who read about nutritionally equivalent compared to nutritionally enhanced cultivated meat. Independent samples t tests indicated that this was the case in both the US (*t*(799) = −0.373, *p* = 0.709) and the UK (*t*(801) = −0.653, *p* = 0.514). The effect sizes confirmed that there was effectively no difference between the two groups in both the US (Cohen’s *d* = 0.033) and the UK (Cohen’s *d* = 0.041).

This is the only major analysis where the trend in the general population differs from that observed in the early majority sample. In the US, the general population was significantly more likely to purchase nutritionally enhanced cultivated meat, but the early majority showed no difference from nutritionally equivalent cultivated meat. It is likely that this difference is accounted for by a ‘ceiling effect’ in the early majority—the sample who read about nutritionally equivalent cultivated meat were already very enthusiastic about it, so there was little to be gained from nutritionally enhanced cultivated meat. However, for the general population, which included consumers who were overall more skeptical, the possibility of nutritional enhancements may be able to act as an additional incentive to consume cultivated meat, at least in the US. This provides an important addition to the conversation on nutritional enhancements of cultivated meat, and the possible effects on consumer acceptance [40].

## 4. Conclusions

This study applied the Diffusion of Innovations framework for understanding the likely future adoption pattern of this new technology and food category—cultivated meat. We assessed adoption in a large representative general population sample in both the US and UK, as well as in the early majority segment and in generational segments. 

Initial population-level support for cultivated meat production was fairly high for an unfamiliar technology (only 5–7% were highly familiar) and this support further increased following reading the expanded description. This increase in support underscores the importance of increasing familiarity with the technology through transparent communication and evidence-based message design, which would be helpful for acceptance of cultivated meat and other novel technologies.

We found that after reading a detailed description of cultivated meat technology, the overall majority were open to supporting the technology, to trying cultivated meat and purchasing it regularly, and to replacing conventional meat, while about half were open to paying more. Openness to cultivated meat increased with the decrease in age of the generational groups. This is in alignment with previous findings [22,53]. Consumers envisioned cultivated meat to be nearly half of their total meat intake. This level of current interest is remarkable for a technology and new food category that is not yet on market.

We assessed consumers’ desired product attributes. Consumers had equal preference for ‘cultivated’ and ‘cultured’ as descriptor terms for the meat product, identifying them as overall more appealing. Despite finding these names slightly less descriptive, consumers nonetheless preferred ‘cultivated’ and ‘cultured’ over ‘cell-based’ and ‘cell-cultured’ in both a social and a labeling context. Assessment of product preferences revealed that government assurances (approval by the FDA and USDA in the US and the FSA in the UK) were the most influential on-package labels, and consumers reported a preference for non-GM cultivated meat. Reasons for adoption were all deemed fairly important by consumers, though consumers in both countries identified cultivated meat’s absence of antibiotics and contribution to global food security as among the highest reasons. In the US, being free from pathogens was of high importance; in the UK, being better for the environment was of high importance. A better nutritional profile made a difference for the general population in the US but not in the UK. These findings have implications for consumer acceptance strategies within these two countries, indicating that messages which highlight individually focused benefits are likely to do better in the US and among late majority groups. 

When examining the early majority segment (the 40% with high interest in trying cultivated meat), nearly all were open to purchasing regularly and three quarters were open to paying more. Measures identifying reasons for adoption were positive in both the general population and the early majority, though the early majority reported these reasons to be more salient. In the nutrition experiment, we found a preference for cultivated meat with better nutrition among the US general population, but no preference was found in the UK or in the early majority groups. Thus, personally beneficial attributes of cultivated meat are not likely to make a great difference for the earlier adopters, but will likely be important for later adopters.

The Diffusion of Innovation framework suggests that adoption of a new innovation typically follows a normal curve over time. With more than 10% of the population indicating a high willingness to pay more, 25–30% highly interested in purchasing regularly, as well as 40% expressing high interest in trying, the results of the study suggest that cultivated meat could become quickly adopted within the general population based on the Diffusion model. 

There are some limitations to this study worth addressing. First, data collection via self-reported online survey is subject to known issues, including data inaccuracies due to imperfect recollection or judgement [54]. Moreover, respondents in the survey would likely be subject to considerable uncertainty about cultivated meat, as many were learning about it for the first time. We attempted to ensure respondent quality by including data quality measures and to ensure some level of knowledge by requiring respondents to spend a minimum amount of time reading the descriptions. That said, the conditions in this survey were quite different from those that consumers experience while shopping.

Future research could use the Diffusion of Innovations framework to examine in more detail the preferences and objections of laggards. While it is most advantageous from a sales perspective to examine earlier adopter segment, it is also useful to understand the perceptions and preferences of more skeptical consumers. Moreover, research could use this approach to explore markets for cultivated meat in other countries around the world, including in Singapore, where cultivated meat is now available to consumers [55].

In conclusion, there are solid consumer markets for cultivated meat in the UK and the US, despite an overall lack of familiarity with the product. Younger generations are the most open to trying cultivated meat, and government seals of approval are considered important. Consumers tend to prefer non-GM cultivated meat, and while nutritional enhancements do not add much to consumer appeal overall, they may be an effective way to provide tangible benefits to more skeptical consumers.

## Figures and Tables

**Table 1:** Representative sampling quotas.

Generation	Age Groups (Interlocked Sex by Age Quotas)	US, *N* = 2018 (Weighted Gen Pop)	UK, *N* = 2034 (Weighted Gen Pop)
Gen Z	18–24	275 (550 before 0.5 weight)	245 (490 before 0.5 weight)
Millennial	25–29, 30–34, 35–39	572	580
Gen X	40–44, 45–49, 50–54	553	574
Boomer	55–59, 60–64, 65–69, 70–74	618	635

Notes: We doubled the quota for Gen Z respondents to ensure a sufficient sub-sample size for the generational analysis. For the full general population and early majority analyses, we then applied a 0.50 weight to the Gen Z respondents to ensure an age-representative sample.

**Table 2:** Brief technology description outcome measures.

RQs	Variable Name	Question	Response Options
2a	Support for the technology	To what degree do you support producing meat in this way?	1 = Not at all supportive 2 = Somewhat supportive 3 = Moderately supportive 4 = Very supportive 5 = Extremely supportive
1a	Prior familiarity	Prior to participating in this study, how familiar were you with this new way of producing meat?	1 = Not at all familiar 2 = Somewhat familiar 3 = Moderately familiar 4 = Very familiar 5 = Extremely familiar
3a	Nomenclature—appeal	To what extent does each of the following names sound appealing? (Cultivated|Cultured|Cell-based|Cell-cultured meat)	1 = Not at all appealing 2 = Somewhat appealing 3 = Moderately appealing 4 = Very appealing 5 = Extremely appealing
3b	Nomenclature—differentiation from conventional meat	To what extent does each of the following names help you tell the difference between this type of meat and conventional meat?	1 = Not at all differentiating 2 = Somewhat differentiating 3 = Moderately differentiating 4 = Very differentiating 5 = Extremely differentiating (Cultivated|Cultured|Cell-cultured|Cell-based)
4a	Nomenclature preferences—social context	Overall, which name would you prefer to use in a SOCIAL CONTEXT, for example when eating dinner with your friends and family? Please drag and drop the names to indicate your order of preference.	Participants ordered name options in order of preference (1–4). (Cultivated|Cultured Cell-cultured|Cell-based)
4b	Nomenclature preferences—product packaging	Overall, which name would you prefer to see on a PACKAGE LABEL, for example when you are buying a product at the grocery store? Please drag and drop the names to indicate your order of preference.	Participants ordered name options in order of preference (1–4). (Cultivated|Cultured|Cell-cultured|Cell-based)

The brief description of the technology introduced this new method of meat production as a food innovation, and highlighted the replication of the biological process which occurs within an animal. Please see Supplementary Materials to view the full description.

**Table 3:** Expanded technology description outcome measures.

RQs	Variable	Question	Response Options
2b, 2c	Support for the technology	To what degree do you support producing meat in this way?	1 = Not at all supportive 2 = Somewhat supportive 3 = Moderately supportive 4 = Very supportive 5 = Extremely supportive
5a, 5b, 5c, 5d	Indicators of dietary adoption	Once cultivated meat has become widely available, how likely are you to … Try cultivated meat? Eat cultivated meat as a replacement for conventional meat? Purchase cultivated meat regularly? Pay a higher price for cultivated meat than conventional meat?	1 = Not at all likely 2 = Somewhat likely 3 = Moderately likely 4 = Very likely 5 = Extremely likely
5e	Indicators of dietary adoption—percentage of predicted intake	Now imagine that cultivated meat has become widely available and affordable. Please roughly estimate the percentage of your meat-intake over the course of a year. Drag the slider to indicate your estimates for each product. The total for both products must equal 100.	Participants dragged sliders to indicate the percentage of predicted intake for each meat type.
6	Reasons for dietary adoption	How important to you are each of the following reasons to replace conventional meat with cultivated meat? Cultivated meat is better for animals. Cultivated meat is better for my health. Cultivated meat is better for the environment. Cultivated meat contains no pathogens. Cultivated meat contains no antibiotics. Cultivated meat contributes to global food security. Cultivated meat is a complementary agricultural system that can help small family farmers continue their way of life.	1 = Not at all important 2 = Somewhat important 3 = Moderately important 4 = Very important 5 = Extremely important
7	Preferences for seals of approval	To what degree would each of the following seals of approval be personally important to you when purchasing cultivated meat? FDA/USDA approved Slaughter-free Halal Kosher Carbon-neutral Produced without antibiotics Non-GMO	1 = Not at all important 2 = Somewhat important 3 = Moderately important 4 = Very important 5 = Extremely important
8	Preference toward genetic engineering	If cultivated meat was genetically modified, how likely would you purchase cultivated meat? If cultivated meat was not genetically modified, how likely would you purchase cultivated meat?	1 = Not at all likely 2 = Somewhat likely 3 = Moderately likely 4 = Very likely 5 = Extremely likely

Note. The expanded technology description explains cell cultivation as similar to plant cultivation, describes the personal and collective benefits of cultivated meat, and shares generally when cultivated meat will be available. At the end of the expanded description, a small graphic was included which caricatures cell sampling, a cultivator, and the cooking of cultivated meat. Please see the Supplementary Materials to view the full expanded technology description.

**Table 4:** Comparison of adoption measures between the US and the UK.

	General Population			Early Adopters		
	US Gen Pop Mean (SD)	UK Gen Pop Mean (SD)	*t*-Test	US Early Adopters Mean (SD)	UK Early Adopters Mean (SD)	*t*-Test
Try	2.95 (1.410)	2.93 (1.426)	*t*(4050) = 0.444, *p* = 0.657	-	-	-
Buy regularly	2.55 (1.337)	2.52 (1.331)	*t*(4050) = 0.828, *p* = 0.408	3.75 (0.992)	3.71 (1.006)	*t*(1601) = 0.962, *p* = 0.336
Replace	2.64 (1.369)	2.59 (1.353)	*t*(4050) = 1.266, *p* = 0.206	3.88 (0.971)	3.80 (1.001)	*t*(1601) = 1.473, *p* = 0.141
Pay more *	1.98 (1.205)	1.86 (1.140)	*t*(4050) = 3.405, *p* = 0.001	2.71 (1.295)	2.51 (1.270)	*t*(1601) = 3.169, *p* = 0.002

Note. * indicates a significant difference between the countries on this measure.

## Supplementary Materials

The following are available online at https://www.mdpi.com/article/10.3390/foods10051050/s1.
