# Supplementary material for: US and UK Consumer Adoption of Cultivated Meat: A Segmentation Study
Source: Foods. 2021 May 11;10(5):1050. doi: 10.3390/foods10051050 (PMC8150824; doi:10.3390/foods10051050)
Supplement: Supplementary file 1 [file foods-10-01050-s001.zip › foods-1189104-supplementary.pdf]

Supplementary Material: Weighted Demographic Tables

Generations

	US General Population		US Early Majority		UK General Population		UK Early Majority	
	n	%	n	%	n	%	n	%
Generation Z (18-24)	275	13.6%	128	15.9%	245	12.0%	129	16.0%
Millennials (25-39)	572	28.4%	263	32.9%	580	28.5%	258	32.1%
Generation X (40-54)	553	27.4%	205	25.6%	574	28.2%	210	26.2%
Baby boomers (55-74)	618	30.6%	205	25.6%	635	31.2%	206	25.7%

Notes on sample sizes: The early majority segments (US, $N = 801$; UK, $N = 803$) were sub-sets of the general population samples (US, $N = 2{,}018$; UK, $N = 2{,}034$). For all analyses not focused on generational differences, Gen Z participants were assigned a 0.50 weight in order for the sample to be representative of age. Due to this weighting, the actual number of participants in the Gen Z category was double the numbers listed here ($n = 549$ for the US and $n = 490$ for the UK). We used this sampling method to allow for two types of analysis: one based on a representative sample and another that segmented by generational category. For the generational analysis, we wanted a sufficient sample size for each generation (about 500 in each category).

Gender

	US General Population		US Early Majority		UK General Population		UK Early Majority	
	n	%	n	%	n	%	n	%
Men	993	49.2%	468	58.5%	1,004	49.4%	466	58.1%
Women	1,025	50.8%	333	41.5%	1,029	50.6%	337	41.9%
Other	-	-	-	-	1	0.1%	-	-

Race/Ethnicity

US Ethnic Categories	US General Population		US Early Majority		UK Ethnic Categories	UK General Population		UK Early Majority	
	n	%	n	%		n	%	n	%
Hispanic, Latino, or Spanish	199	9.8%	86	10.7%	Asian/Asian British - Bangladeshi	15	0.7%	5	0.3%
White or Caucasian	1,474	73.1%	617	77.0%	Asian/Asian British - Chinese	22	1.1%	10	0.6%
Black or African American	289	14.3%	97	12.1%	Asian/Asian British - Indian	44	2.2%	18	1.1%
American Indian or Alaskan Native	68	3.4%	31	3.9%	Asian/Asian British - Pakistani	25	1.2%	10	0.6%
Asian	84	4.1%	39	4.9%	Asian/Asian British - Other	24	1.2%	11	0.7%
South Asian (Indian Subcontinent)	13	0.6%	4	0.5%	Black - African	39	1.9%	12	0.7%
Middle Eastern or North African	15	0.7%	8	1.0%	Black - Caribbean	32	1.6%	12	0.7%
Native Hawaiian or Other Pacific Islander	5	0.2%	3	0.3%	Black - Other	3	0.1%	2	0.1%
Other	17	0.8%	6	0.7%	White - British	1,635	80.4%	742	46.3%
Prefer not to say	7	0.3%	2	0.2%	White - Irish	28	1.4%	12	0.7%
					White - Gypsy or Irish Traveler	3	0.1%	1	0.1%
					White - Other	141	6.9%	55	3.4%
					Other - Arab	8	0.4%	2	0.1%
					Other - Any other ethnic group	20	1.0%	7	0.4%
					Prefer not to say	17	0.8%	4	0.2%

Region

	US General Population		US Early Majority			UK General Population		UK Early Majority	
US Regions	n	%	n	%	UK Regions	n	%	n	%
Northeast	336	16.7%	135	16.8%	Northern Ireland	59	2.9%	24	3.0%
Midwest	514	25.6%	218	27.3%	Scotland	170	8.4%	74	9.2%
South	735	36.7%	255	31.9%	North East	75	3.7%	35	4.4%
West	420	21.0%	192	24.0%	North West	234	11.6%	98	12.2%
					Yorkshire & The Humber	172	8.5%	61	7.6%
					West Midlands	175	8.6%	66	8.2%
					East Midlands	175	8.6%	70	8.8%
					Wales	102	5.0%	42	5.3%
					East Anglia	150	7.4%	58	7.3%
					London	258	12.7%	105	13.1%
					South West	160	7.9%	62	7.7%
					South East	298	14.7%	107	13.4%

Population Density

	US General Population		US Early Majority		UK General Population		UK Early Majority	
	n	%	n	%	n	%	n	%
Rural area or village	364	18.0%	124	15.5%	327	16.2%	122	15.1%
Small town	342	17.0%	128	16.0%	500	24.7%	189	23.5%
Middle-sized town	602	30.0%	248	31.0%	440	21.7%	170	21.1%
Large town or city	691	34.5%	299	37.4%	751	37.1%	317	39.4%

Don’t know	6	0.3%	2	0.3%	7	0.3%	3	0.4%

Education Levels

	US General Population		US Early Majority		UK General Population		UK Early Majority	
	n	%	n	%	n	%	n	%
No education	2	0.1%	2	0.2%	5	0.2%	2	0.3%
Primary school	2	0.1%	-	-	7	0.3%	3	0.3%
Some high school	64	3.2%	19	2.3%	64	3.1%	24	3.0%
Completed high school	681	34.0%	272	34.0%	469	23.2%	154	19.3%
Technical qualification or trade certificate	259	12.9%	96	12.0%	577	28.5%	221	27.7%
College/ undergraduate degree	767	38.2%	312	39.0%	693	34.2%	306	38.2%
Postgraduate degree	231	11.5%	100	12.5%	211	10.4%	90	11.3%

Annual Income Levels

	US General Population		US Early Majority		UK General Population		UK Early Majority	
	n	%	n	%	n	%	n	%
Less than $20,000	343	17.1%	119	14.8%	598	29.6%	210	26.3%
$20,000 to $39,999	458	22.8%	176	22.0%	685	33.9%	265	33.2%
$40,000 to $59,999	382	19.0%	161	20.2%	428	21.2%	189	23.7%
$60,000 to $79,999	287	14.3%	111	13.9%	169	8.3%	70	8.8%
$80,000 to $99,999	183	9.1%	74	9.2%	88	4.4%	37	4.6%
$100,000 or more	353	17.6%	159	19.9%	55	2.7%	28	3.4%

Religion

	US General Population		US Early Majority		UK General Population		UK Early Majority	
	n	%	n	%	n	%	n	%
Hinduism	10	0.5%	3	0.4%	23	1.1%	8	1.0%
Islam	18	0.9%	4	0.4%	66	3.2%	22	2.7%
Christianity	1,203	59.6%	448	55.9%	907	55.8%	313	39.1%
Sikhism	1	0.1%	-	-	5	0.2%	3	0.3%
Buddhism	19	0.9%	9	1.1%	13	0.6%	4	0.5%
Jainism	1	0.1%	1	0.1%	1	0.1%	1	0.1%
Judaism	37	1.8%	17	2.1%	13	0.6%	3	0.4%
Taoism	2	0.1%	1	0.1%	1	0.1%	1	0.1%
Confucianism	1	0.1%	-	-	1	0.1%	-	-
Other religion	181	9.0%	56	6.9%	57	2.8%	19	2.4%
Agnostic/Atheist/ Spiritual /Not religious	531	26.5%	261	32.6%	940	46.4%	426	53.3%

Political Orientation

	US General Population		US Early Majority		UK General Population		UK Early Majority	
	n	%	n	%	n	%	n	%
Very liberal	192	9.6%	106	13.3%	142	7.0%	74	9.3%
Liberal	389	19.4%	191	23.9%	427	21.1%	216	27.0%
Moderate	829	41.4%	288	36.0%	940	46.5%	333	41.6%
Conservative	367	18.3%	130	16.2%	448	22.1%	150	18.7%
Very conservative	228	11.4%	85	10.6%	68	3.3%	28	3.4%

Dietary Patterns

		US General Population		US Early Majority		UK General Population		UK Early Majority	
		n	%	n	%	n	%	n	%
Diet	Vegan	24	1.2%	4	0.4%	27	1.3%	6	0.7%
	Vegetarian	47	2.3%	11	1.4%	92	4.5%	21	2.6%
	Pescatarian	62	3.1%	21	2.6%	76	3.7%	23	2.8%
	Omnivore	1,879	93.4%	764	95.4%	1,833	90.5%	752	93.8%
Meat consumption frequency	None	71	3.5%	16	1.9%	138	6.8%	29	3.6%
	Light	309	15.4%	105	13.1%	610	30.1%	227	28.4%
	Medium	987	49.1%	420	52.6%	1088	53.7%	443	55.3%
	Heavy	643	32.0%	259	32.4%	191	9.4%	103	12.8%

Supplementary Material: Stimuli

Brief Technology Description

Real meat—produced directly from cells

One recent breakthrough in food innovation is meat production without raising and slaughtering animals. This new method of meat production mirrors the biological process of building muscle but does so outside of the animal and under controlled conditions.

A small sample of starter cells grows into genuine meat—the same as it would inside the animal—when it is given essential nutrients like proteins, vitamins, and minerals. The final product looks, cooks, and tastes the same.

Expanded Technology Description

Meat—cultivated directly from cells

We can now diversify and strengthen the protein supply by producing meat in a new, more efficient way. Rather than raising and slaughtering animals, we can cultivate meat directly. This starts with the basic building block of all life—the cell.

A few years ago, the first hamburger was prepared from meat cultivated directly from cow cells. By isolating the cells needed to produce meat and enabling them to grow under controlled conditions, researchers demonstrated that it is possible to produce genuine beef outside of a cow.

In fact, we can grow not only beef but also pork, poultry, and seafood without needing to farm pigs, chickens, and fish. Instead, we can use what is known as a cultivator.

The cultivator facilitates the same biological process that happens inside an animal by providing warmth and the basic elements needed to build muscle: water, proteins, carbohydrates, fat, vitamins, and minerals. Cultivating meat is similar to growing plants from cuttings in a greenhouse, which provides warmth, fertile soil, water, and nutrients.

This new method of meat production enables the natural process of cell growth but in a more efficient environment. The result is an abundance of cultivated meat, identical to conventional meat at the cellular level but free from pathogens and trace contaminants such as antibiotics. Cultivated meat looks, cooks, and tastes the same.

Cultivated meat companies are aiming to produce real meat that offsets the need to raise animals in intensive confinement systems. [In the United States, 99% of conventional meat is produced in industrial confinement systems and 1% is produced on smallholder farms.]

Compared with conventional meat production, meat cultivation is less resource-intensive, decreasing methane emissions, deforestation, biodiversity loss, water use, water pollution, antibiotic resistance, and foodborne illnesses.

Food companies and universities around the world are working on scaling this method of meat production and bringing cultivated meat to market in the next 2-3 years. [The FDA and the USDA will jointly regulate cultivated meat in the United States.]

This will expand the protein options available to consumers, providing the meat so many people desire, simply produced in a new and more sustainable way.

Note: In the UK survey, the above bracketed sentences were replaced respectively with the following sentences: 1) In the United Kingdom, 73% of conventional meat is produced in industrial confinement systems and 27% is produced on smallholder farms. 2) The FSA will regulate cultivated meat in the United Kingdom.

Graphic Following Expanded Technology Description

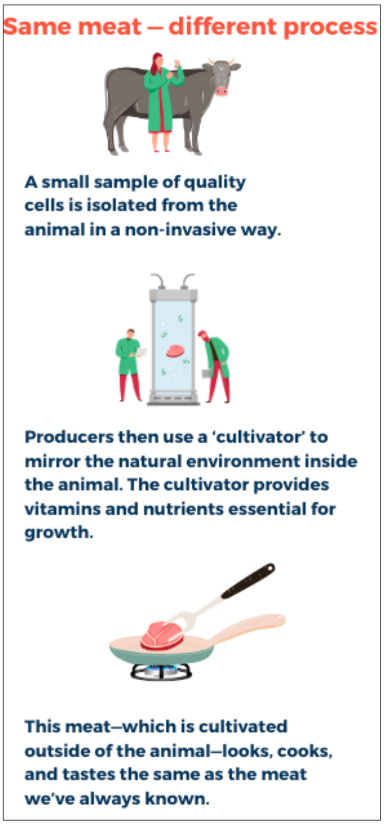

Nutrition Experimental Message

Same Nutritional Profile	Better Nutritional Profile
The steak shown in this photo is thin-cut cultivated steak prepared for a culinary demonstration.	The steak shown in this photo is thin-cut cultivated steak prepared for a culinary demonstration.

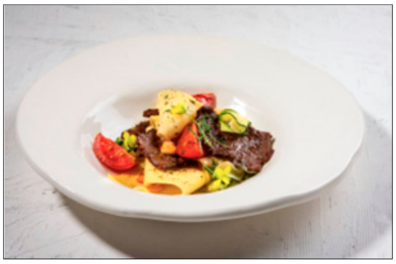

The cultivated steak in this photo tastes the same as conventionally produced steak.

Producers cultivate beef to have the same nutritional profile than conventional beef:

- Nutritious aspects of beef—like vitamins and minerals such as B12 and iron—are the same.
- Non-nutritious aspects of meat—including saturated fat and cholesterol—are the same.

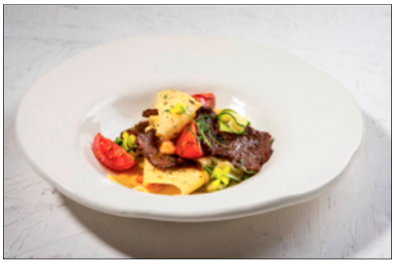

The cultivated steak in this photo tastes the same as conventionally produced steak.

Producers cultivate beef to have the better nutritional profile than conventional beef:

- Nutritious aspects of beef—like vitamins and minerals such as B12 and iron—are higher.
- Non-nutritious aspects of meat—including saturated fat and cholesterol—are lower.
